# O.4.4-2 Cross-sectoral sporting programs aimed at children from low-income families: lessons learnt from the Netherlands

**DOI:** 10.1093/eurpub/ckad133.199

**Published:** 2023-09-11

**Authors:** Linda Ooms, Kirsten Gutter, Caroline van Lindert

**Affiliations:** Mulier Institute, The Netherlands; l.ooms@mulierinstituut.nl; Mulier Institute, The Netherlands; l.ooms@mulierinstituut.nl; Mulier Institute, The Netherlands; l.ooms@mulierinstituut.nl

## Abstract

**Purpose:**

Children from low-income families participate less often in sport and physical activity than children from high-income families. In the Netherlands, sporting programs were implemented by sports clubs in collaboration with other local organizations to stimulate physical activity among children from poor families. These programs were funded by the Dutch government within the program ‘Sport Impulse Youth from Low Income Neighborhoods’ (2014-2020). The aim of the research was to get insight into the implementation and successfulness of these sporting programs with regard to increasing sport participation among the target group.

**Methods:**

After the two-year funding period, online questionnaires were sent to project coordinators and group interviews were conducted with project coordinators and collaborating partners (funding period 2014-2017). During the final funding period (2018-2020), also group interviews were conducted with participating children and parents. The research focused on the reach and implementation of the programs, involvement of parents, experienced effects, and factors influencing sport participation among the target group.

**Results:**

Sporting programs were often adapted to the local context. Furthermore, there was little collaboration with key figures in the neighborhood that were close to the target group. The target group was predominantly reached through providing sport activities at schools in low-income neighborhoods. Providing different sport activities and personal guidance, stimulated children’s participation. However, a lack of follow-up activities and a lack of parental involvement, hindered structural sport participation.

**Conclusions:**

The target group can be reached with these kind of sporting programs. To enhance program effectiveness, it is important to involve parents and to ensure suited follow-up activities are available. Furthermore, it is important that the sport coach is someone who is familiar with the background of the target group. Moreover, collaborations with key figures in the neighborhood could help in attracting more participants. Finally, adaptations in sporting programs should be thoroughly monitored.

**Funding source:**

This study was funded by ZonMw, The Netherlands.

